# Accelerating Clinical Evaluation of Repurposed Combination Therapies for COVID-19

**DOI:** 10.4269/ajtmh.20-0995

**Published:** 2020-08-21

**Authors:** Craig R. Rayner, Louis Dron, Jay J. H. Park, Eric H. Decloedt, Mark F. Cotton, Vis Niranjan, Patrick F. Smith, Michael G. Dodds, Fran Brown, Gilmar Reis, David Wesche, Edward J. Mills

**Affiliations:** 1 Monash Institute of Pharmaceutical Sciences, Monash University, Melbourne, Australia;; 2 Certara, Princeton, New Jersey;; 3 Cytel Inc., Vancouver, Canada;; 4 Department of Health Research Methods, Evidence, and Impact, McMaster University, Hamilton, Canada;; 5 Department of Medicine, Experimental Medicine, University of British Columbia, Vancouver, Canada;; 6 Division of Clinical Pharmacology, Department of Medicine, Faculty of Medicine and Health Sciences, Stellenbosch University, Cape Town, South Africa;; 7 Family Clinical Research Unit, Department of Paediatrics and Child Health, Faculty of Medicine and Health Sciences, Stellenbosch University, Cape Town, South Africa;; 8 RxMD, Chennai, India;; 9 PUC Minas Medical School of Medicine at Contagem, Belo Horizonte, Brazil

## Abstract

As the global COVID-19 pandemic continues, unabated and clinical trials demonstrate limited effective pharmaceutical interventions, there is a pressing need to accelerate treatment evaluations. Among options for accelerated development is the evaluation of drug combinations in the absence of prior monotherapy data. This approach is appealing for a number of reasons. First, combining two or more drugs with related or complementary therapeutic effects permits a multipronged approach addressing the variable pathways of the disease. Second, if an individual component of a combination offers a therapeutic effect, then in the absence of antagonism, a trial of combination therapy should still detect individual efficacy. Third, this strategy is time saving. Rather than taking a stepwise approach to evaluating monotherapies, this strategy begins with testing all relevant therapeutic options. Finally, given the severity of the current pandemic and the absence of treatment options, the likelihood of detecting a treatment effect with combination therapy maintains scientific enthusiasm for evaluating repurposed treatments. Antiviral combination selection can be facilitated by insights regarding SARS-CoV-2 pathophysiology and cell cycle dynamics, supported by infectious disease and clinical pharmacology expert advice. We describe a clinical evaluation strategy using adaptive combination platform trials to rapidly test combination therapies to treat COVID-19.

For novel COVID-19, there has been extensive focus on repurposing previously approved drugs against SARS-CoV-2.^1^ Repurposing is attractive, as it allows the use of existing information on human pharmacology and clinical safety to enable faster clinical trial development and rollout.^2^ In addition, repurposing drugs offers the potential to rapidly and efficiently scale effective treatments, in contrast to newer therapies that require upscaling drug manufacturing and supply chain pathways.

To illustrate, a recent large-scale compound repurposing effort identified more than 20 antivirals that should be further investigated for application for COVID-19.^3^ Antiviral agents for COVID-19 can be categorized by two broad mechanisms of action: 1) those targeting viral proteins or nucleic acids related to infection of host cells, viral production via hijacking cellular machinery, or release from host cells and circulation of virions; and 2) those targeting essential host functions for viral replication including agents such as interferon that boost the cellular immune response to infection. Each category is complex, and of the armament of potential repurposed drugs for COVID-19, multitudes of mechanisms and pathways are in consideration. Selectively combining agents to complement each other by variable mechanisms of action across these categories may yield effective treatments for select phases of SARS-CoV-2 infection. Analogous to the evolution of antiretroviral therapies for HIV infection, it is important to formulate and test combination therapy regimens. Instead of sequentially testing monotherapies that will likely have modest clinical effects on their own, additive or synergistic effects can potentially be gained by combining antiviral drugs exploiting pharmacology throughout the spectrum of COVID-19 illness.^4^

There are more than 750 clinical trials evaluating repurposed therapies for COVID-19.^1^ These trials cover all antiviral mechanisms noted earlier and applications from prophylaxis to treatment to reduction in the sequelae of the host inflammatory response. However, to date, only dexamethasone and remdesivir have been identified as effective therapies for severely ill patients.^5,6^ This low success rate might be due to the fact that the majority of COVID-19 clinical trials (87%) are evaluating repurposed drugs as monotherapy.^1^

Combination therapies for COVID-19 represent an attractive approach to drug development. Among the first trials published of effective interventions for COVID-19 was a combination of interferon beta-1b, lopinavir–ritonavir (LPV/r), and ribavirin for hospitalized patients.^7^ In comparison to the control group that received LPV/r monotherapy, the triple combination arm showed faster viral clearance and alleviation of symptoms, and shorter hospital stays.^7^ Although LPV/r monotherapy was no better than placebo alone in another hospitalized trial (RECOVERY),^8^ it is unclear whether both interferon beta-1b and ribavirin or triple combination therapy drove clinical benefits for hospitalized patients.

## ESTABLISHING COMBINATION THERAPIES

Under normal circumstances, development of combination treatment strategies entails a stepwise evaluation process whereby first the individual components of a potential combination regimen are tested for clinical efficacy in isolation or as individual arms within a trial evaluating both single and combination regimens. This strategy allows sequential compilation of evidence for each drug before studying combinations, as outlined by the U.S. Food and Drug Administration in describing the so-called combination rule.^9^

This process is based on reducing exposure to ineffective or toxic drugs among participants until individual components have demonstrated treatment effects. Although this process is both scientifically rigorous and safe, it is slow, and may delay identification of unexpected synergistic effects, resulting in failure to evaluate potentially potent treatment cocktails.

## ACCELERATING COMBINATION THERAPIES

As the global pandemic continues, unabated and therapeutic options evaluated to date demonstrate limited effectiveness, and there is a pressing need to accelerate treatment evaluations. Among the options for accelerated development is the evaluation of combination strategies in the absence of prior monotherapy data. This approach is appealing for a number of reasons. First, combining two or more drugs with complementary antiviral or therapeutic effects permits a multipronged approach addressing the variable pathways of the disease. Second, if an individual component of a combination strategy offers a therapeutic effect, then the clinical trial should still detect treatment effects unless antagonism between components is present. Third, this strategy saves time, as it accelerates evaluation of prioritized combination regimens much earlier than would occur with a stepwise approach evaluating monotherapies before combinations. Finally, given the severity of the current pandemic, and the current absence of verified treatment options, the likelihood of detecting a treatment effect with combination therapy maintains scientific and patient enthusiasm for repurposed treatments.

Despite these advantages, there has been limited research activity investigating combination therapy for COVID-19, in clear contrast to other disease areas. Among the 1971 registered clinical trials evaluating all therapeutic interventions (i.e., not only previously approved treatments) for COVID-19,^1^ only 258 (13.1%) trials are evaluating combination therapies. Of these, 120 trials are taking a stepwise approach, and 138 trials are evaluating combination strategies without associated individual monotherapies. Determining whether these trials are implementing a forward (evaluating monotherapy before combination therapy) or backward stepwise approach is not possible from clinical trial registries.

## TRIAL DESIGN FOR EVALUATING COMBINATIONS AND THEIR COMPONENTS

Among the many scientific lessons of the COVID-19 pandemic has been the widespread embrace of adaptive clinical trials, particularly platform trials in which multiple interventions are compared simultaneously against a common control arm, and in which interventions may be added over time.^10,11^ Although this concept is not new, it has historically been controversial and limited to industry-run clinical trials, especially in oncology. In adaptive clinical trial designs, prespecified modifications are permitted with decision rules based on accumulated interim data.^12^ Adaptive designs have been controversial because interim data are often assessed multiple times, leading to a fear of inflated type I error rates. However, statistical measures can be implemented to control the type I error rate at the usual 5%. Important examples of adaptive platform trials directed toward COVID-19 include the RECOVERY,^5^ SOLIDARITY,^13^ and REMAP-CAP^14^ trials.

**Figure 1.**
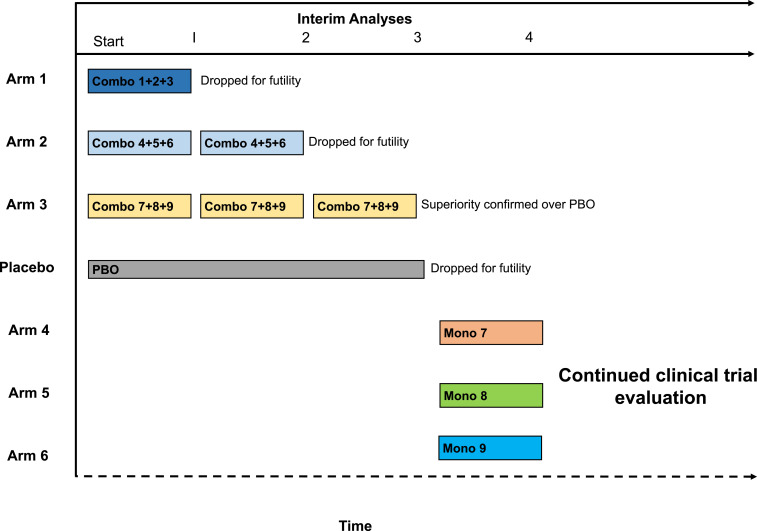
A platform trial for combination therapy. Here, in this example, there are several interim analyses planned for the platform trial testing combination therapies using a backward stepwise approach. At the first interim analysis, combination arm 1 is dropped for futility followed by combination arm 2 dropped at the second interim analysis. At the third interim analysis, combination arm 3 shows superiority over placebo (PBO), and, thereafter, individual monotherapies are added and evaluated after.

Adaptive platform trials provide capacity in combination trials to add or remove ineffective treatment arms, allowing for sequential comparisons and a perpetual design wherein treatments can be prioritized or de-prioritized as evidence accumulates. We propose a platform trial to evaluate combination therapies in the absence of monotherapy evaluations (Figure 1). If the combination is ineffective, then there is reduced incentive to evaluate the monotherapies. If the combination demonstrates efficacy, then monotherapy arms can be studied to determine which components of the combination therapy are efficacious.

Our proposed approach is not without limitations. Evaluating combinations assumes that individual components are nonantagonistic and likely either additive or synergistic. There are important examples of drug therapies that exhibit antagonism.^15,16^ Thus, combination strategies may miss efficacy of individual components undermined by antagonism. Furthermore, drug interactions may result in adverse events and toxicity not observed with monotherapy. Therefore, it is critical to engage clinical pharmacologists and infectious disease experts in guiding the selection and refinement of regimens to ensure that insights on SARS-CoV-2 pathophysiology, cell cycle dynamics, mechanisms of action, drug–drug interactions, safety signals, and clinical utility are appropriately incorporated in the selection of combinations for study.

Ultimately, we argue that the best approach to quickly establish efficacy of potential therapies for COVID-19 is through studying combination therapies, ideally with demonstrated in vitro and in vivo activities and strong preclinical properties, in an adaptive framework to maximize the probability of clinical efficacy. This strategy challenges the traditional drug development dogma, but we believe that it will accelerate the development of repurposed drugs as combination therapies to treat COVID-19.
